# Hydroxysafflor Yellow A Alleviates Acute Myocardial Ischemia/Reperfusion Injury in Mice by Inhibiting Ferroptosis via the Activation of the HIF-1α/SLC7A11/GPX4 Signaling Pathway

**DOI:** 10.3390/nu15153411

**Published:** 2023-07-31

**Authors:** Chaowen Ge, Yuqin Peng, Jiacheng Li, Lei Wang, Xiaoyu Zhu, Ning Wang, Dongmei Yang, Xian Zhou, Dennis Chang

**Affiliations:** 1 Anhui Province Key Laboratory of Research & Development of Chinese Medicine, Anhui University of Chinese Medicine, Hefei 230012, China; gcwedu@163.com (C.G.); pyqedu@163.com (Y.P.);; 2 Anhui Province Key Laboratory of Chinese Medicinal Formula, Anhui University of Chinese Medicine, Hefei 230012, China; 3 Institute for Pharmacodynamics and Safety Evaluation of Chinese Medicine, Anhui Academy of Traditional Chinese Medicine, Hefei 230012, China; 4 Anhui Medical College, Hefei 230601, China; zhuxy1119@foxmail.com; 5 NICM Health Research Institute, Western Sydney University, Westmead, Sydney, NSW 2145, Australia

**Keywords:** HSYA, myocardial ischemia/reperfusion injury, ferroptosis, HIF-1α/SLC7A11/GPX4

## Abstract

Ferroptosis is closely associated with the pathophysiology of myocardial ischemia. Hydroxysafflor yellow A (HSYA), the main active ingredient in the Chinese herbal medicine safflower, exerts significant protective effects against myocardial ischemia/reperfusion injury (MI/RI). The aim of this study was to investigate the protective effects of HSYA against MI/RI and identify the putative underlying mechanisms. An in vivo model of acute MI/RI was established in C57 mice. Subsequently, the effects of HSYA on myocardial tissue injury were evaluated by histology. Lipid peroxidation and myocardial injury marker contents in myocardial tissue and serum and iron contents in myocardial tissue were determined using biochemical assays. Mitochondrial damage was assessed using transmission electron microscopy. H9C2 cardiomyocytes were induced in vitro by oxygen–glucose deprivation/reoxygenation, and ferroptosis inducer erastin was administered to detect ferroptosis-related indicators, oxidative-stress-related indicators, and expressions of ferroptosis-related proteins and HIF-1α. In MI/RI model mice, HSYA reduced myocardial histopathological damage, ameliorated mitochondrial damage in myocardial cells, and decreased total cellular iron and ferrous ion contents in myocardial tissue. HSYA increased the protein levels of SLC7A11, HIF-1α, and GPX4 and mitigated erastin- or HIF-1α siRNA-induced damage in H9C2 cells. In summary, HSYA alleviated MI/RI by activating the HIF-1α/SLC7A11/GPX4 signaling pathway, thereby inhibiting ferroptosis.

## 1. Introduction

Coronary heart disease (CHD), also known as “ischemic heart disease,” is characterized by stenosis or obstruction of the arterial lumen resulting from an atherosclerotic lesion of the coronary artery. Myocardial infarction is the leading cause of death among ischemic heart disorders [1]. In the ischemic–hypoxic state, the myocardium undergoes further pathological changes in response to reperfusion, mainly including Ca^2+^ overload, disturbances in mitochondrial energy metabolism, oxidative stress, inflammation, and ferroptosis, which are referred to as myocardial ischemia/reperfusion injury (MI/RI) [2]. Clinical treatment drugs are mainly used to dilate blood vessels and reduce reperfusion injury, such as nifedipine and so on [3,4]. However, these drugs often cause some adverse reactions. Therefore, finding new drugs has become an urgent clinical problem to be solved [5].

Ferroptosis is a novel nonapoptotic and iron-dependent cell death mode that originates from two compounds: erastin and RSL3 [6]. In 2003, a small molecule named erastin was found to induce a new nonapoptotic cell death process [7]. This cell death inhibits the uptake of glutathione (GSH) through the cystine/glutamate antiporter (SystemXc-) system [8]. Ferroptosis is characterized by a buildup of cellular iron, the promotion of lipid peroxidation, and a reduction in antioxidant defenses. Morphologically, ferroptosis is typified by cell enlargement, rupture of the plasma membrane, mitochondrial contraction, and an increase in mitochondrial membrane density [9]. Importantly, ferroptosis also represents a key mechanism underlying ischemia/reperfusion (I/R)-induced cardiomyopathy [10].

Studies have shown that the disruption of the System x_c_^−^/GSH/GPX4 antioxidant system is the key mechanism behind the induction of ferroptosis. System x_c_^−^ is a cystine–glutamate antiporter localized to the membranes of mammalian cells. It consists of two subunits, namely, a light chain (solute carrier family 7 member 11, SLC7A11), which primarily controls the activity of the antiporter, and a heavy chain (solute carrier family 3 member 2, SLC3A2) [11].

System x_c_^−^ is mainly responsible for the balance of amino acids inside and outside the cell. Cystine entering the cell is reduced to cysteine, an essential raw material for the synthesis of glutathione (GSH) [12]. GSH is a cellular antioxidant that can effectively eliminate intracellular free radicals and maintain cellular redox homeostasis and exists in dynamic balance with oxidized glutathione (GSSH) under the action of glutathione peroxidase 4 (GPX4). GPX4 converts toxic polyunsaturated fatty acids (PUFA-OOH) into nontoxic alcohol forms (PUFA-OH), effectively preventing lipid peroxide accumulation in cells.

Hypoxia-inducible factor 1 alpha (HIF-1α) is a transcription factor that mediates the adaptive response of cells to ischemia and/or hypoxia and plays a critical role in the maintenance of oxygen homeostasis by regulating the transcriptional activity of a wide range of target genes. Studies have demonstrated that HIF-1α can exert myocardial protective effects by attenuating oxidative stress, autophagy, the inflammatory response, and apoptosis caused by I/R [13]. The inhibition of HIF-1α expression was reported to exacerbate ferroptosis induced by sorafenib [14]. Additionally, it has been shown that HIF-1α can enhance SLC7A11 mRNA stability via the upregulation of lncRNA-PMAN [15]. However, whether HIF-1α is involved in the regulation of SLC7A11/GPX4-mediated ferroptosis during myocardial I/R remains unknown.

Hydroxysafflor yellow A (HSYA) is the main active ingredient in safflower and has been reported to exert significant ameliorative effects in MI/RI. The key mechanisms behind the protective effects of HSYA include the inhibition of calcium influx, apoptosis, inflammation, mitochondrial malfunction, and activation of autophagy, as well as the amelioration of oxidative stress [16]. One study showed that HSYA stimulates the tube-forming and migratory ability of human umbilical vein endothelial cells and upregulates the expression of nucleolin, VEGF-A, and matrix metalloproteinase 9 (MMP-9) [17]. HSYA also restores mitochondrial energy metabolism, reduces reactive oxygen species (ROS) production, and inhibits apoptosis by activating the PI3K/AKT/hexokinase II pathway, thereby suppressing chronic hypoxia-induced apoptosis [18]. Additionally, HSYA attenuates calcium overload in cardiomyocytes after I/R injury and significantly increases Ca^2+^ uptake by calcium depots [19]. Combined, these observations indicate that HSYA can alleviate MI/RI by scavenging oxygen free radicals and attenuating lipid peroxidation.

In this study, we explored whether the ameliorative effects of HSYA in MI/RI are associated with the regulation of ferroptosis and whether this involves the HIF-1α/SLC7A11/GPX4 signaling pathway by employing a C57/BL6J mouse model of MI/RI and an H9C2 cell model of OGD/R.

## 2. Materials and Methods

### 2.1. Reagents and Chemicals

HSYA (BH20968, source leaf purity >98%) and nifedipine (NIF) tablets were obtained from Tianjin Pacific Pharmaceutical Co., Ltd. (Tianjin, China). PX-478 (SJ-MX0279; HIF-1α inhibitor), ferrostatin-1 (SJ-MX0049; ferroptosis inhibitor), erastin (SJ-MX0039; ferroptosis inducer), creatine kinase (CK, JL11280), creatine kinase myocardial band (CK-MB, JL18284), cardiac troponin I (CTNI, JL12296), creatine phosphokinase (CPK, JL11280), lactate dehydrogenase (LDH, JL20461), lipid peroxide (LPO, JL13877), and GPX4 (JL20370) ELISA kits were purchased from Shanghai Jianglai Biotechnology Co., Ltd. (Shanghai, China). 4-Hydroxynonenal (4-HNE; MM-0796R1) was purchased from Jiangsu Meimian Industrial Co., Ltd. (Yangzhou, China). Anti-GPX4 (ab125066), anti-HIF-1α (ab179483), and Abcam. anti-SLC7A11 (ab216876) fluorescent antibodies were purchased from the USA. Anti-HIF-1α antibody (340462), anti-SLC7A11 antibody (382036), and goat antirabbit IgG secondary antibody (511203) were obtained from Chengdu Zen BioScience Co., Ltd. (Chengdu, China). Antifluorescence quenching blocker (P0126) was obtained from Shanghai Biyuntian Biotechnology Co., Ltd. (Shanghai, China). iCell-R012 (H9C2) was obtained from Sebachem (Shanghai, China). Fetal bovine serum (10270106) and DMEM (C11995500BT) were purchased from Gibco. The Cellular Iron Assay Kit (BestBio, Shanghai, China, BB-47511) was obtained from Shanghai BestBio Biotechnology Co., Ltd. Lipid peroxidation fluorescent probe (MKBio, Shanghai, China, MX5211) and ferrous ion fluorescent probe (MKBio, Shanghai, China, MX4580) were purchased from Shanghai Maokang Biotechnology Co., Ltd. (Shanghai, China).

### 2.2. Mouse Model of Myocardial Ischemia/Reperfusion Injury and Groupings

Seventy mature male C57BL/6 mice (21–22 g) were purchased from Henan Skbex Biotechnology Co., Ltd. (Anyang, China). After one week of acclimatization feeding, the mice were randomly assigned to one of the following seven treatment groups (n = 10 per group): a sham operation group, a MIRI group, three HSYA groups (5, 10, or 20 mg/kg), a positive drug control group (NIF 10 mg/kg), and an HIF-1α inhibitor group (PX-478 10 mg/kg). NIF was given by gavage once daily (at 08:00 h) for two weeks. HSYA was administered intraperitoneally once daily (at 08:00 h) for two weeks. PX-478 was administered intraperitoneally twice weekly for two weeks [20]. On day 14, a mouse model of MI/RI was established 1 h after drug administration via the ligation of the left anterior descending coronary artery (LAD). Once full anesthesia had been established (intraperitoneal injection of 50 mg/kg sodium pentobarbital), a catheter was inserted into the trachea and connected to a ventilator. The left side of the thorax of the mouse was shaved and routinely disinfected. An incision of approximately 1 cm was made near the edge of the sternum, and the heart was exposed at the fourth intercostal space. The LAD was temporarily ligated with 6-0 nylon monofilament (Golden Circle, Shanghai, China) for 30 min. LAD occlusion was confirmed, and the model was successfully established when blood flow laser speckle imaging showed left ventricular ischemia. After 30 min of local myocardial ischemia, the sutures were loosened, and LAD reperfusion was then performed for 120 min. After reperfusion, the animals were euthanized, and the relevant samples were collected for analysis. The experimental design was approved by the Experimental Animal Ethics Committee of the Anhui University of Chinese Medicine (ethical lot number: AHUCM-mouse-2023004).

### 2.3. Infarct Area Assessment

A continuous-wavelength (λ = 785 nm) laser source was used to illuminate the observed area of the heart exposed during reperfusion vertically, which was reflected by biological tissues and collected through an internal CCD imaging system. Due to the divergence through different optical paths, the emitted light interferes with itself to form a random interference pattern that is processed by the computer to form a false color map, which is clearly displayed. The direction of blood flow and blood perfusion in the heart of the sham group and the MIRI group were compared. The volume of the myocardial infarct was determined using 2,3,5-triphenyl tetrazolium chloride TTC and Evans blue (EB) staining. After reperfusion, the LAD was reoccluded after injection of 0.2 mL of 2% EB solution via the right internal jugular vein. The heart was removed, rinsed three times with precooled PBS, and sectioned. The heart was stained with TTC at 37 °C for 15–20 min. After staining, the normal myocardium was blue, the I/R injury area was brick red, and the infarcted myocardium was pale white. The sections were then fixed in 4% paraformaldehyde for 24 h and photographed. Image analysis was performed using ImagePro Plus 6.0.

### 2.4. Histological Analysis

Heart tissues were postfixed in 4% paraformaldehyde, washed in saline solution, embedded in paraffin, and sliced into 4 mm thick sections. The specimens were subjected to hematoxylin and eosin (H&E) and Masson staining using standard protocols. Histopathological changes were assessed under a microscope.

### 2.5. ELISA

The levels of CK, CK-MB, CTNI, CPK, LDH, LPO, GPX4, and 4-HNE in myocardial tissue, blood samples, and H9C2 cell supernatant were detected using the double-antibody sandwich method. All the indicators were detected with the corresponding kits. The absorbance value was measured by a microplate reader at a wavelength of 450 nm, and the content of each indicator in the samples was calculated by using the standard curve.

### 2.6. GSH, MDA, and Iron Measurements

Total iron, GSH, and MDA concentrations in myocardial tissue were determined by colorimetric assay according to the manufacturer’s instructions.

### 2.7. Transmission Electron Microscopy

Cardiac tissue or cells were precipitated, fixed with an electron microscopy fixative, dehydrated through a graded ethanol series, embedded, sliced into 50–80 nm thick sections using an ultramicrotome, and stained with 5% uranyl acetate and lead citrate. The sections were then observed and imaged using a Hitachi transmission electron microscope (TEM, JEM-1400, Jeol, Tokyo, Japan).

### 2.8. Immunofluorescence

The expression of HIF-1α, SLC7A11, and GPX4 in myocardial tissues was detected by immunofluorescence using antibodies targeting them. The secondary antibody was goat antirabbit IgG (1:5000). Images were captured using a laser scanning confocal microscope. The fluorescence intensity of the target proteins was determined with ImageJ.

### 2.9. Western Blotting

The total protein extracted from cardiac tissue was separated by SDS–PAGE and transferred to PVDF membranes. After 2 h of treatment with 5% skim milk at room temperature, three washes in trisbuffered saline Tween-20 (TBST), and overnight incubation with the primary antibody at 4 °C, the membrane was sealed. The detected proteins and their corresponding dilutions were as follows: HIF-1α (1:500), SLC7A11 (1:500), GPX4 (1:500), or actin (1:5000). After rinsing, the membrane was incubated with an HRP-labeled secondary antibody (1:10,000) for 1 h at 37 °C in a constant temperature oscillation incubator. The protein bands were developed using an ECL kit and analyzed using ImageJ software.

### 2.10. Cell Culture and Treatment

To mimic ischemia in vitro, oxygen–glucose deprivation and reoxygenation (OGD/R) were applied in H9C2 cells. The cells were grown in DMEM without glucose in an anaerobic environment with 94% N_2_, 5% CO_2_, and 1% O_2_ for 3 h and then reoxygenated for 6 h under typical culture conditions with DMEM containing 10% FBS. To detect the protective effect of HSYA, H9C2 cells were divided into a normal control group, an OGD/R group, three HSYA groups (HSYA 1.25, 5, or 20 μmol/L), and a positive control drug group (NIF 100 μmol/L). To investigate whether HSYA exerted an inhibitory effect on lipid peroxidation in H9C2 cells, the following groups were established: a normal control group, an OGD/R group, an HSYA group (HSYA 20 μmol/L), a Fer-1 group (Fer-1 10 μmol/L), an HSYA+Fer-1 group (HSYA 20 μmol/L+Fer-1 10 μmol/L), an erastin group (Erastin 5 μmol/L), and an HSYA + Erastin group (HSYA 20 μmol/L + Erastin 5 μmol/L).

### 2.11. Cell Viability Assay

Cell viability was assessed using a CCK-8 kit following the manufacturer’s instructions, in which H9C2 cells were seeded in 96-well plates and allowed to grow to 80% confluence. Following OGD/R, HSYA was applied to the cells for 6 h. Subsequently, 10 µL of CCK-8 reagent in 100 µL of culture medium was added to each well for 2 h at 37 °C. The optical density (OD) of each well was determined at 450 nm using a microplate reader.

### 2.12. ROS, Lipid Peroxidation, and Fe^2+^ Assay

ROS contents were assessed by fluorescence microscopy using a DCFH-DA oxidation-sensitive fluorescent probe. Lipid peroxidation and Fe^2+^ accumulation were detected by flow cytometry using the C11 BODIPY581/591 molecular probe and the FerroFarRed fluorescent probe.

### 2.13. HIF-1α-Targeted RNA Interference and Transfection

At 80% confluence, H9C2 cardiomyocytes were transfected with small interfering RNAs (siRNAs) targeting HIF-1α according to the manufacturer’s instructions (Guangzhou RIBOBIO Co., Ltd., Guangzhou, China). The sequences were as follows (5′–3′): GACTCAGTTTGAACTAACT, CTCCCTATATCCCAATGGA, and TCGACAAGCTTAAGAAAGA. Twenty-four hours after transfection, HIF-1α protein expression levels were assessed using immunoblotting.

### 2.14. Statistical Analysis

Statistical analysis was conducted using GraphPad Prism 8.0.2. The data are presented as means ± SDs. The data were tested for normality before applying parametric statistics using the Shapiro–Wilk normality test. The equality of variances was tested using the Brown–Forsythe test. For comparisons between two groups, a two-tailed Student’s *t*-test was applied for normal data, in which *p* < 0.05 was chosen as the cutoff for statistical significance.

## 3. Results

### 3.1. The Surgical Model of MI/RI Was Tested Using Laser Speckle Imaging of Blood Flow

**Figure 1:**
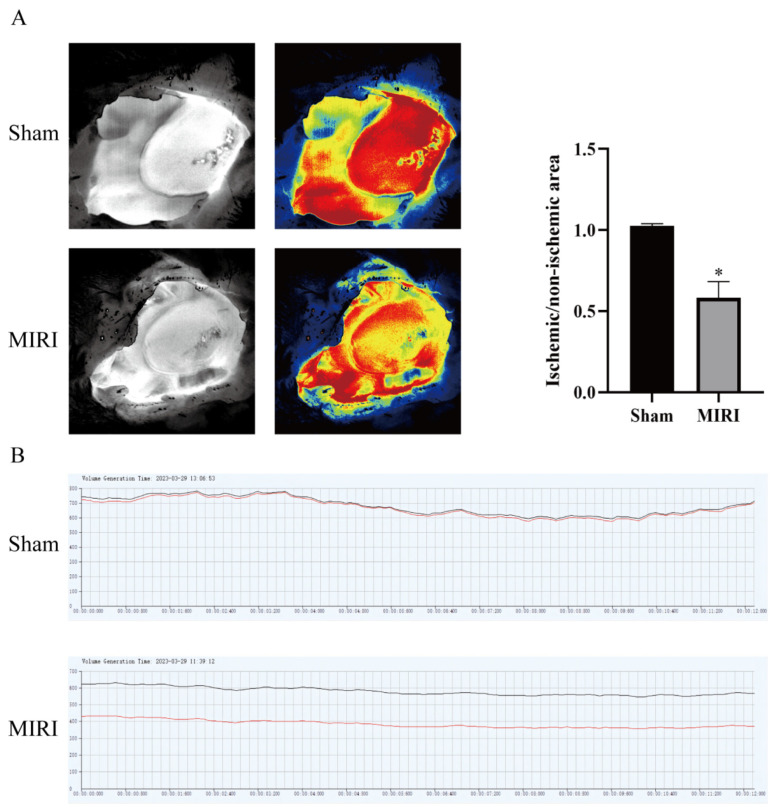
The myocardial ischemia model was tested by blood flow laser speckle imaging. (**A**) Pseudocolor diagram of the basic state and typical state of myocardial ischemia/reperfusion (*n* = 3). (**B**) Changes in blood flow between the basic state and the typical state of myocardial ischemia/reperfusion (*n* = 3). * *p* < 0.05 vs. the sham operation group. Black line is the blood flow of the normal group and the red line is the blood flow of the model group.

To confirm that the MI/RI model had been established, the mice were treated with HSYA for 14 days and then submitted to MI/RI. The procedure was considered successful when a significant reduction in perfusion in the myocardial infarct area was observed compared with that in the noninfarct area, followed by the detection of reperfusion (Figure 1A,B).

### 3.2. The Protective Effect of HSYA on MI/RI in Mice

**Figure 2:**
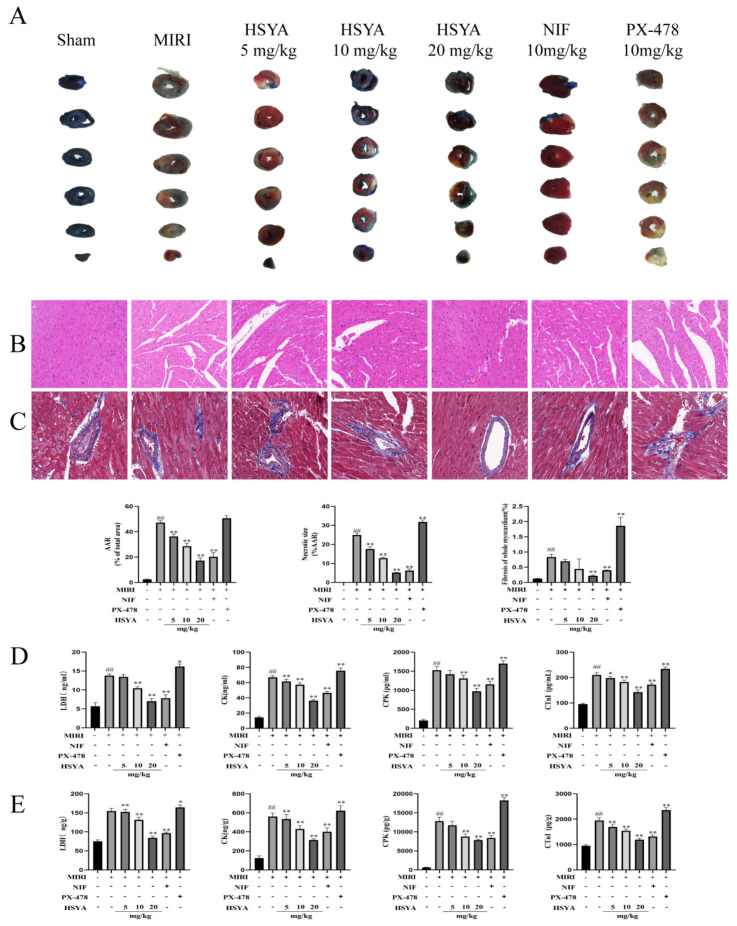
Hydroxysafflor yellow A (HSYA) alleviated pathological injury in mice. (**A**) TTC staining was used to detect the area of myocardial infarction (*n* = 3). (**B**) Hematoxylin and eosin staining was used to detect myocardial injury (×400) (*n* = 3). (**C**) Masson staining was used to detect myocardial injury (×400) (*n* = 3). ## *p* < 0.01 vs. the sham group, ** *p* < 0.01 vs. the MI/RI group. (**D**,**E**) The expression of LDH, CK, CPK, and CTNI in myocardial tissue and serum was determined by ELISA (*n* = 6). ^##^ *p* < 0.01 vs. the sham group; * *p* < 0.05, ** *p* < 0.01 vs. the MI/RI group.

To evaluate the protective effect of HSYA on MI/RI in mice, TTC/EB double staining was used to observe the myocardial tissue infarct volume after treatment (Figure 2A). In the figure, the blue area is the normal area, the remaining area (including red and white) is the ischemic risk area (AAR), where the white area is the myocardial infarction area (IA), and the red area is the ischemic but not infarcted area. The myocardial ischemic area was calculated as the percentage of AAR in the left ventricle, and the myocardial infarction area was calculated as the percentage of IA in the AAR. The extent of myocardial injury was detected by H&E and Masson staining. As shown in Figure 2B,C, the myocardial tissue structure of mice in the control group was normal, without edema, lesions, or neutrophil infiltration. Myocardial injury was severe in the MI/RI mice, as characterized by the presence of inflammation and interstitial edematous lesions in the myocardial tissue. However, treatment with either HSYA or NIF improved the above pathological injury to some extent, with more significant effects observed with high-dose HSYA treatment. These results suggested that HSYA can reduce morphological changes in the myocardium following I/R injury. Additionally, the pathological damage was more extensive in the PX-478 group than in the MI/RI-only group. ELISA was used to evaluate the levels of LDH, CK, CPK, and CTNI in both serum (Figure 2D) and cardiac tissue (Figure 2E). Animals in the MI/RI group had considerably higher levels of LDH, CK, CPK, and CTNI than those in the sham group. Similarly, compared with the MI/RI group, the levels of these proteins were markedly lower in the NIF and HSYA groups, with the best results seen in the HSYA high-dose group. This implied that HSYA could minimize myocardial injury to some extent. Meanwhile, LDH, CK, CPK, and CTNI levels were considerably higher in mice of the PX-478 group than in those of the MI/RI-only group. Combined, these results indicate that HSYA exerts protective effects against MIRI in mice, whereas inhibiting HIF-1α has the opposite effect.

### 3.3. HSYA Alleviates Lipid Peroxidation Induced by MI/RI in Mice

**Figure 3:**
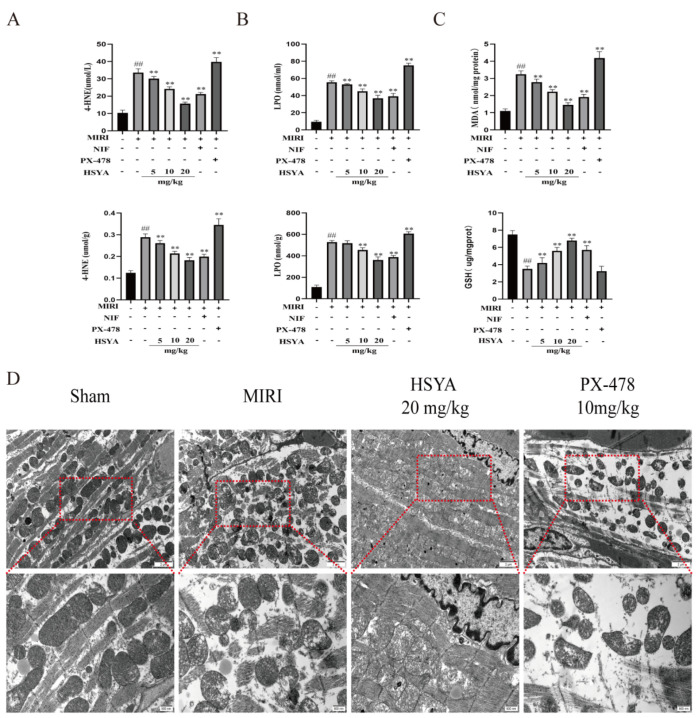
Hydroxysafflor yellow A (HSYA) ameliorated myocardial ischemia/reperfusion injury (MI/RI)-induced lipid peroxidation and mitochondrial damage in myocardial tissue. (**A**,**B**) 4-HNE and LPO in myocardial tissue and serum levels were assessed using the respective ELISA kits (*n* = 6). (**C**) MDA content and GSH expression in myocardial tissue were detected using the respective kits (*n* = 6). ^##^ *p* < 0.01 vs. the sham operation group, ** *p* < 0.01 vs. the MI/RI group. (**D**) The effect of HSYA on MI/RI-induced mitochondrial damage in mice was observed by TEM (×25,000).

ELISA was used to detect 4-HNE and LPO levels in both serum and myocardial tissue. The results showed that the levels of both proteins were abnormally elevated in the myocardial tissue (Figure 3A) and serum (Figure 3B) of MI/RI mice compared with those of mice in the sham operation group and, to a lesser extent, those in the HSYA and NIF groups. We further measured the levels of oxidative-stress-related indexes in the mice following the respective treatments. We found that the contents of MDA were lower in the HSYA group (Figure 3C) than in the MI/RI group, where MDA content is an important indicator of the degree of lipid peroxidation, whereas GSH levels displayed the opposite trend. In the PX-478 group, 4-HNE, LPO, and MDA levels were increased, whereas those of GSH were decreased compared with that seen in the MI/RI-only group. These results indicated that HSYA mitigated MI/RI-induced lipid peroxidation, whereas the inhibition of HIF-1α expression exacerbated this effect. Given that mitochondria play a central role in cell growth and ROS generation, we next observed mitochondrial ultrastructure in the myocardium of the various treatment groups using TEM analysis. The results showed that MI/RI induced mitochondrial swelling and a reduction in the number of mitochondrial cristae, or even their disappearance. In addition, significant improvements in myofilament breakage were observed in the ultrastructure of myocardial tissue in the HSYA group (Figure 3D).

### 3.4. Iron Accumulation in Cardiac Tissue

**Figure 4:**
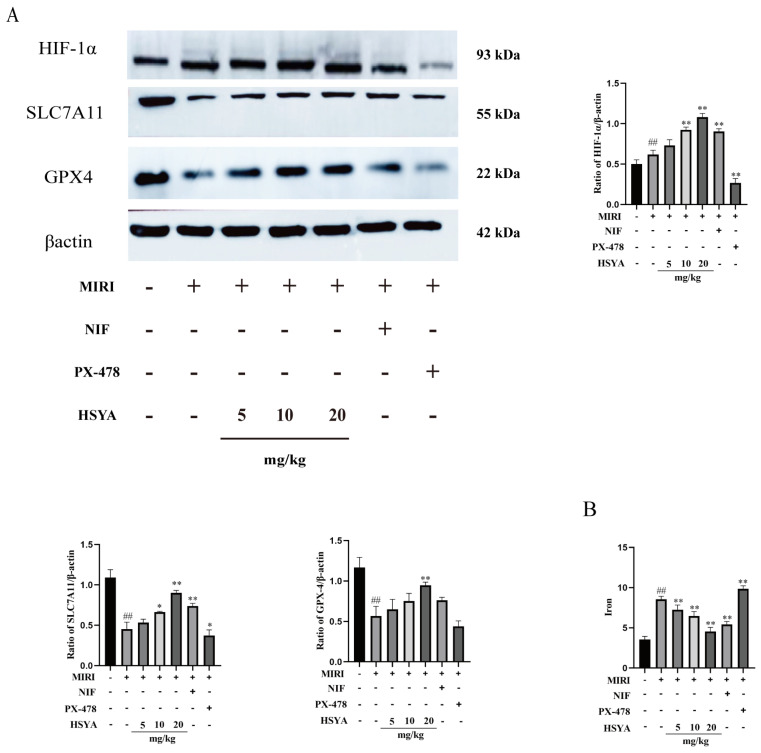
Hydroxysafflor yellow A alleviated ferroptosis by activating the HIF-1α/SLC7A11/GPX4 signaling pathway. (**A**) The levels of ferroptosis-related proteins were assessed by Western blotting (*n* = 3). (**B**) The total iron content in cardiac tissue was determined using a kit (*n* = 6). ^##^ *p* < 0.01 vs. the sham group; * *p* < 0.05, ** *p* < 0.01 vs. the MI/RI group.

The expression of ferroptosis-related proteins was detected by Western blotting (Figure 4A). The results showed that the expression of HIF-1α was significantly higher and that of SLC7A11 and GPX4 significantly lower in the MI/RI group compared with that in the sham operation group. Compared with the MI/RI group, HIF-1α, SLC7A11, and GPX4 expression was increased in both the HSYA and NIF groups, with the best effect detected with high-dose HSYA treatment. SLC7A11 and GPX4 expression levels were lower in the PX-478 group than in the HSYA group. Finally, iron levels in cardiac tissue were found to be considerably higher in the MI/RI and PX-478 groups than in the HSYA groups (Figure 4B). These observations suggest that HSYA can reduce iron buildup in mice following MI/RI.

### 3.5. Measurement of ROS Levels in H9C2 Cells

**Figure 5:**
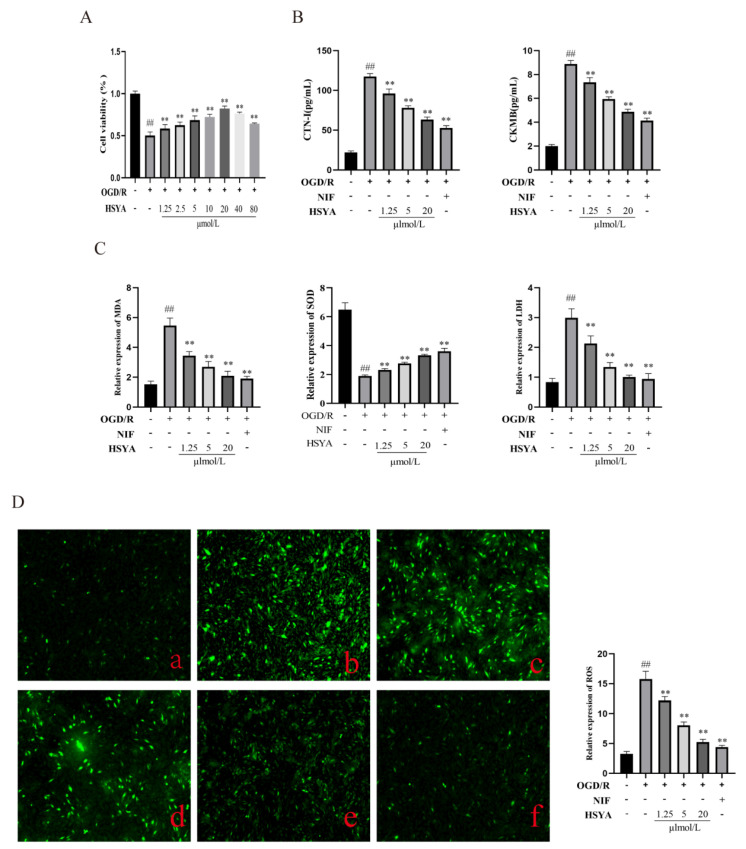
Hydroxysafflor yellow A (HSYA) improved oxygen–glucose deprivation/reoxygenation (OGD/R)-induced injury in H9C2 cells. (**A**) Cell viability was detected using a Cell Counting Kit-8 (*n* = 6). (**B**) The expression of CTNI and CKMB was detected by ELISA (*n* = 6). (**C**) MDA, SOD, and LDH levels were detected using the respective kits (*n* = 6). (**D**) Reactive oxygen species (ROS) levels were measured using DCFH-DA staining: (**a**) Control group; (**b**) OGD/R group; (**c**–**e**) HSYA (1.25, 5, 20 μmol/L); (**f**) nifedipine (NIF; *n* = 3). ^##^ *p* < 0.01 vs. the control group, ** *p* < 0.01 vs. the OGD/R group.

H9C2 cells subjected to OGD/R displayed significantly reduced cell viability and increased levels of ROS, indicative of oxidative stress. HSYA and NIF (100 μmol/L, as per [15]) have been shown to exert protective effects against myocardial I/R injury in vitro. Here, we examined the proliferative ability (Figure 5A) and the contents of MDA, SOD, and LDH and found that they were significantly improved after HSYA treatment (Figure 5C) in H9C2 cells and that the maximum concentration of HSYA that was not cytotoxic to the cells was 20 μmol/L. SOD is one of the important indicators reflecting the metabolic state of free radicals in the human body, which plays a crucial role in the balance between oxidation and antioxidation in the body. The level of SOD can indirectly reflect the ability of the body to remove free radicals. The levels of CTNI and CKMB in the supernatants of the different treatment groups were determined by ELISA (Figure 5B). The HSYA groups effectively improved the abnormally elevated levels of CKMB and CTNI after myocardial injury. Furthermore, compared with the model group, ROS generation was reduced in the HSYA(1.5, 5, 20 μmol/L) groups.

### 3.6. HSYA Protects against Mitochondrial Damage in Cardiomyocytes by Inhibiting Lipid Peroxidation

**Figure 6:**
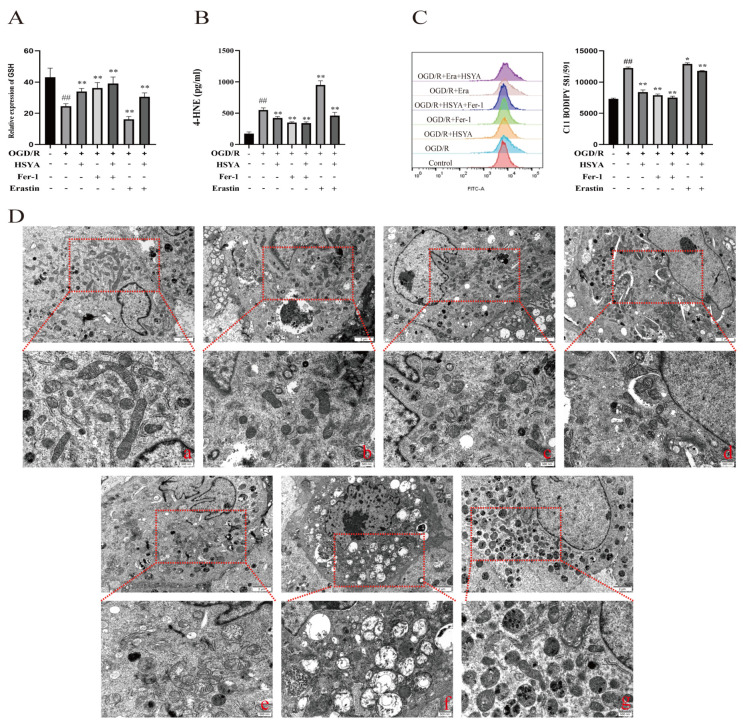
Hydroxysafflor yellow A (HSYA) improved oxygen–glucose deprivation/reoxygenation (OGD/R)-induced lipid peroxidation and mitochondrial damage in H9C2 cells. (**A**) The GSH level was detected using a kit (*n* = 6). (**B**) The expression of 4-HNE was detected by ELISA (*n* = 6). (**C**) The effect of HSYA on OGD/R- and erastin-induced lipid peroxidation (*n* = 3). (**D**) Transmission electron micrographs showing mitochondrial morphology in H9C2 cells (×25,000): (**a**) Sham group; (**b**) OGD/R group; (**c**) OGD/R+HSYA group; (**d**) OGD/R+Fer-1 group; (**e**) OGD/R+HSYA+Fer-1 group; (**f**) OGD/R+Erastin group; (**g**) OGD/R+Erastin+HSYA group. ^##^ *p* < 0.01 vs. the control group; * *p* < 0.05, ** *p* < 0.01 vs. the OGD/R group.

To observe the effect of HSYA on oxidative stress levels, lipid peroxidation, and mitochondrial morphology, H9C2 cells were subjected to OGD/R and then treated with the ferroptosis inhibitor Fer-1 (10 μmol) [21] or the ferroptosis inducer erastin (5 μmol) [22]. The results showed that both OGD/R and erastin treatment induced marked damage in H9C2 cells. However, compared with OGD/R treatment, the administration of either high-dose HSYA or Fer-1 effectively reduced the levels of ferroptosis, increased the GSH content (Figure 6A), and decreased the 4-HNE content (Figure 6B) in H9C2 cells, thereby reducing oxidative stress levels and enhancing the oxygen-free-radical scavenging ability of the cells. In addition, flow cytometric analysis showed that HSYA reduced lipid peroxide levels in H9C2 cells (Figure 6C). We further evaluated the extent of changes in mitochondrial morphology in H9C2 cells and found that both HSYA and Fer-1 treatments effectively increased mitochondrial cristae numbers, reduced mitochondrial swelling, and increased mitochondrial membrane density (Figure 6D).

### 3.7. HSYA Reduces Iron Accumulation in Cardiac Muscle Cells

**Figure 7:**
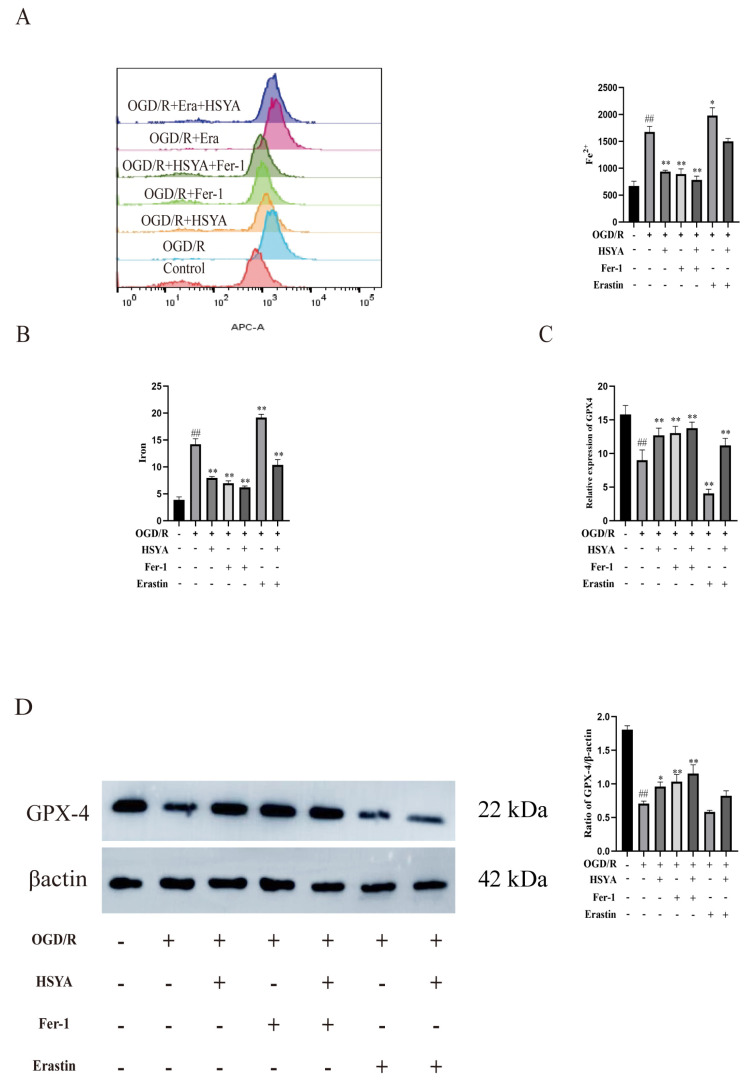
Hydroxysafflor yellow A inhibited oxygen–glucose deprivation/reoxygenation (OGD/R)-induced ferroptosis in H9C2 cells. (**A**) The Fe^2+^ level was detected by flow cytometry (*n* = 3). (**B**) The total iron content in cells was detected using a kit (*n* = 6). (**C**) The expression of GPX4 was evaluated by ELISA (*n* = 6). (**D**) The expression of the ferroptosis-related protein GPX4 was measured by Western blot (*n* = 3). ^##^ *p* < 0.01 vs. the control group; * *p* < 0.05, ** *p* < 0.01 vs. the OGD/R group.

We also assessed the effects of HSYA on iron accumulation in H9C2 cells. The results showed that Fe^2+^ (Figure 7A) and total iron (Figure 7B) contents were significantly higher in the OGD/R and OGD/R+Erastin groups than in the HSYA group. We further investigated the effect of HSYA on GPX4 levels in these cells and found that HSYA or Fer-1 treatment reversed the decrease in GPX4 levels induced by OGD/R or OGD/R+Erastin (Figure 7C), as determined by Western blot analysis (Figure 7D). These results indicated that HSYA reduced iron accumulation induced by OGD/R or OGD/R+Erastin treatment in H9C2 cells.

### 3.8. The Inhibition of HIF-1α Expression Promoted Ferroptosis in H9C2 Cells

**Figure 8:**
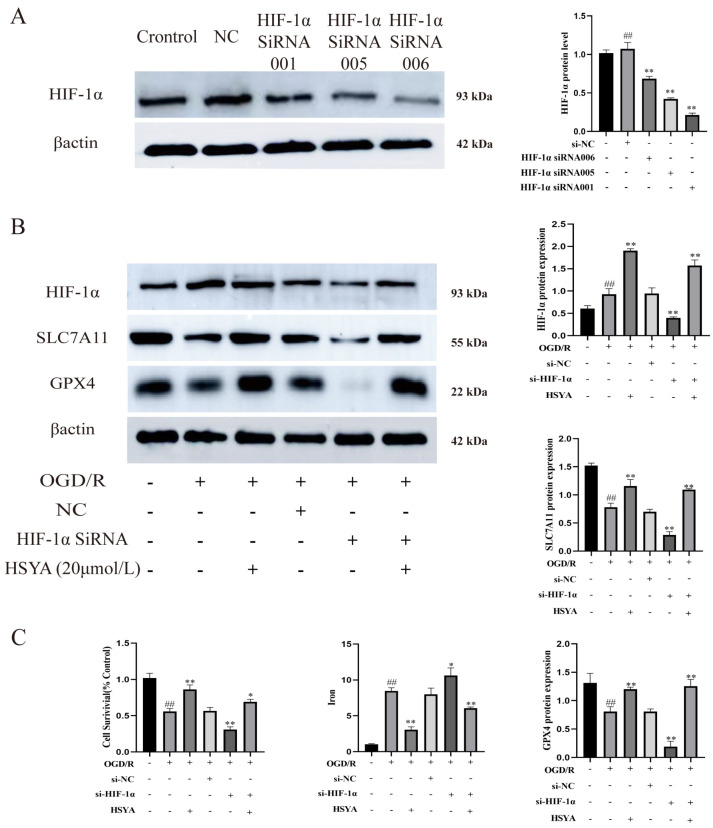
The inhibition of HIF-1α expression promoted ferroptosis. (**A**) HIF-1α protein levels were measured by Western blot (*n* = 3). ^##^ *p* < 0.01 vs. the control group, ** *p* < 0.01 vs. the normal control (NC) group. (**B**) The expression of ferroptosis- and HIF-1α-related proteins was detected by Western blot (*n* = 3). (**C**) Cell viability and total iron contents were determined using the respective kits (*n* = 6). ^##^ *p* < 0.01 vs. the control group; * *p* < 0.05, ** *p* < 0.01 vs. the OGD/R group.

Finally, HIF-1α expression was depleted in H9C2 cells by transfection with siRNA targeting HIF-1α (Figure 8A). The expression of HIF-1α, SLC7A11, and GPX4 was significantly reduced in HIF-1α siRNA-treated cells, as shown in Figure 8B. Therefore, we selected the most effective HSYA (20 μmol/L) group among the screened doses. However, HSYA administration improved the viability of H9C2 cells in the OGD/R+HIF-1α siRNA group and reduced iron accumulation in these cells (Figure 8C).

## 4. Discussion

Clinical results show that myocardial ischemia–reperfusion injury can produce a large amount of ROS, resulting in lipid peroxidation damage. Therefore, scavenging oxygen free radicals and reducing lipid peroxidation damage are important measures to improve myocardial ischemia–reperfusion injury [23].

HSYA, the primary ingredient in safflower, exerts multiple effects in the treatment of cerebral and myocardial I/R injury. It reduces the generation of ROS [24], promotes angiogenesis [25], inhibits apoptosis [26], and protects mitochondrial function against oxidative damage. HSYA has been shown to promote neovascularization and the recovery of cardiac function through the HO-1/VEGF-A/SDF-1α cascade [27]. The mechanisms by which HSYA protects against MI/RI are known to include the inhibition of NLRP3 inflammasome activation and the promotion of autophagy [28]. However, ours is the first study to report that the ameliorative effects of HSYA on MI/RI may be mediated, at least in part, via the inhibition of ferroptosis.

Ferroptosis is a nonapoptotic mode of cell death characterized by the iron-dependent accumulation of lipid peroxidation to lethal levels—effects that are associated with a wide variety of neurological and ischemic diseases [29]. Multiple pathological factors or drugs can lead to the activation of the mitochondrial-dependent anion channel and mitogen-activated protein kinase signaling [30], endoplasmic reticulum stress, and the inhibition of the function of the cystine/glutamate antiporter (System x_c_^−^) in cells. This results in an increase in intracellular iron uptake and the intensification of the iron-dependent Fenton reaction [31]. It has been shown that the disruption of the System x_c_^−^/GSH/GPX4 antioxidant axis is the primary mechanism behind the induction of ferroptosis. For example, a reduction in the expression of the key associated proteins—SLC7A11 and GPX4—has been reported to reduce the antioxidant capacity of cells and lead to ferroptosis. However, supplementing the normal amount of dietary iron does not cause ferroptosis.

In this study, we found that HSYA significantly mitigated the damage to myocardial tissue in MI/RI model mice. Total myocardial iron accumulation and MDA levels were significantly reduced, mitochondrial damage was alleviated, ROS and lipid peroxidation levels were reduced, and the expression of ferroptosis-related proteins was increased, as evidenced by immunofluorescence analysis. To determine whether HIF-1α regulates ferroptosis during MI/RI in mice, we administered the HIF-1α inhibitor PX-478 and found that myocardial histopathological damage, iron accumulation, and lipid peroxidation were significantly increased. This suggested that HSYA may improve MI/RI in mice by activating HIF-1α and thereby inhibiting ferroptosis.

In an H9C2 cell model of OGD/R, HSYA treatment significantly decreased the levels of CKMB, CPK, CTNI, MDA, and LDH in the supernatant, while SOD activity was significantly increased. The administration of HSYA to OGD/R model H9C2 cells treated with the ferroptosis inducer erastin led to a significant reduction in iron accumulation and lipid peroxidation levels, suggesting that HSYA may protect H9C2 cells from OGD/R damage by inhibiting ferroptosis.

H9C2 cells depleted of HIF-1α via siRNA transfection exhibited a marked reduction in the expression of the ferroptosis-related proteins SLC7A11 and GPX4. However, HSYA administration upregulated the contents of HIF-1α, SLC7A11, and GPX4; reduced total iron levels; and greatly increased the viability of H9C2 cells. These observations suggest that the HSYA-mediated activation of HIF-1α induces the expression of SLC7A11 and GPX4 and further imply that activating HIF-1α may exert a significant regulatory effect on ferroptosis during acute myocardial I/R.

## 5. Conclusions

**Figure 9:**
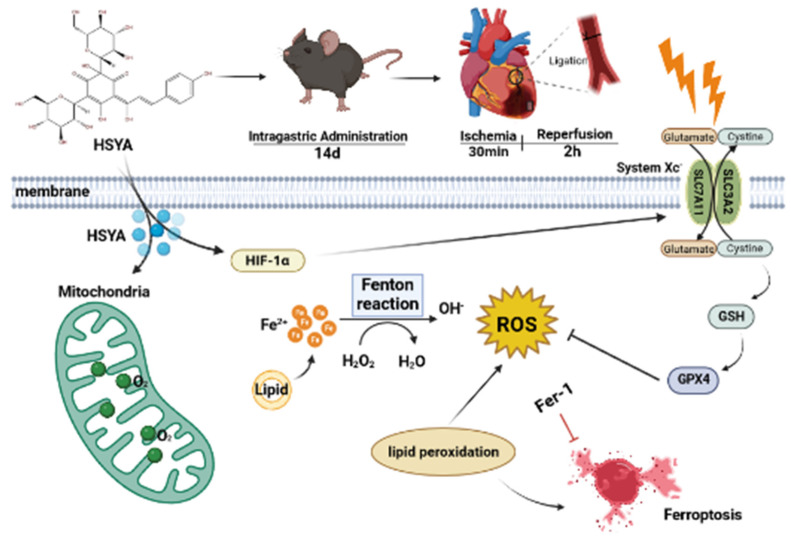
Schematic overview of the mechanism underlying the protective effects of hydroxysafflor yellow A (HSYA) against myocardial ischemia/reperfusion injury (MI/RI)-induced ferroptosis in H9C2 cells.

In this study, we showed that the HIF-1/SLC7A11/GPX4 signaling pathway may mediate the protective effects of HSYA on MI/RI in mice, at least in part (Figure 9). Our study provides a reference and scientific basis for the clinical study of the mechanism and medication of MI/RI.

## Data Availability

Data presented in this study are available on request from the corresponding author.

## Abbreviations

4-HNE, 4-Hydroxynonenal; CCK-8, Cell Counting Kit-8; CHD, coronary atherosclerotic heart disease; CK, creatine kinase; CKMB, creatine kinase myocardial band; CPK, creatine phosphokinase; CTNI, cardiac troponin I; EB, Evans blue; GPX4, glutathione peroxidase 4; GSH, glutathione; H&E, hematoxylin and eosin; HIF-1α, hypoxia-inducible factor 1; HSYA, hydroxysafflor yellow A; LDH, lactate dehydrogenase; LPO, lipid peroxide; MDA, malondialdehyde; MI/RI, myocardial ischemia/reperfusion injury; OGD/R, oxygen–glucose deprivation/reperfusion; SOD; superoxide dismutase; TEM, transmission electron microscopy; TTC, 2,3,5-Ttriphenyl tetrazolium chloride.
